# Factors influencing Thai university students’ decisions to take COVID-19 vaccine booster doses: a cross-sectional survey

**DOI:** 10.1186/s41182-024-00597-1

**Published:** 2024-04-17

**Authors:** Weerakorn Thichumpa, Naphat Yimthin, Anawat Ratchatorn, Satoko Izumi, Wirichada Pan-ngum

**Affiliations:** 1 Department of Tropical Hygiene, Faculty of Tropical Medicine, Mahidol University, Bangkok, Thailand; 2 School of Integrated Health Sciences, Faculty of Medicine, The University of Tokyo, Tokyo, Japan; 3 Mahidol Oxford Tropical Medicine Research Unit, Faculty of Tropical Medicine, Mahidol University, Bangkok, Thailand

**Keywords:** COVID-19 vaccine, Vaccine acceptance, Booster doses, Public health

## Abstract

**Background:**

We aimed to describe the acceptance of COVID-19 vaccine booster doses and factors influencing this among Thai university students.

**Methods:**

A cross-sectional survey was conducted between July and September 2022. All university students in Thailand were eligible to participate. We explored the acceptance rate of COVID-19 vaccine booster doses and regular vaccines (if available) among university students. Associations between factors influencing the acceptance of vaccination were analyzed by multiple logistic regression analysis.

**Results:**

A total of 322 participants were surveyed (78.9% female, age 18 to 49 years (mean = 22.6, standard deviation = 5.47)). Most participants (85.7%) were undergraduate students (Bachelor level), and a proportion (84.8%) had a background in health sciences studies. The proportions who accepted booster doses and regular vaccines were 52.8% and 69.3%, respectively. Vaccine accessibility was found to be significantly associated with the acceptance of booster doses (adjusted odds ratio (AOR) = 2.77, 95% confidence interval (CI) = 1.10–6.97), while the availability of scientific evidence (AOR = 3.44, 95% CI = 1.21–9.77) was significantly associated with the acceptance of regular vaccines.

**Conclusions:**

This study contributes to addressing the knowledge gap regarding acceptance of COVID-19 vaccine booster doses among university students in Thailand. Our findings revealed that vaccine accessibility and the availability of scientific evidence, as well as vaccination costs, influenced individuals’ decisions around accepting vaccine booster doses. Further research should focus on the dynamics of vaccine acceptance to facilitate the development of targeted strategies and support vaccination policymaking in Thailand.

## Background

Coronavirus disease (COVID-19) is an infectious respiratory illness caused by the SARS-CoV-2 virus. The impact of COVID-19 on human health globally has been huge, with widespread transmission observed [1, 2]. In 2022 there have been more than 700 million confirmed cases and approximately 6.6 million deaths worldwide [2]. To address the urgent need to curb the spread and severity of this infection, various preventive strategies have been implemented, and the World Health Organization (WHO) has approved several vaccines for use against COVID-19 [2]. According to previous studies and recommendations, COVID-19 vaccines have demonstrated strong efficacy in preventing severe illness, hospitalization, and death [3–5]. Administering vaccine booster doses has emerged as a promising approach to reduce the risk of COVID-19 infection and counter the waning of neutralizing antibodies that occurs following the initial vaccine regimen [6]. Substantial evidence supports the role of COVID-19 vaccination in reducing disease transmission and enhancing herd immunity [7, 8]. The Center of Disease prevention and Control (CDC) recommends five- or six-month booster doses (Pfizer or Moderna) after completing the primary course of COVID-19 vaccine [9]. However, vaccine hesitancy persists among some individuals who have concerns regarding vaccine safety, efficacy, and potential side effects [10].

Thailand has been grappling with the COVID-19 situation since the first quarter of 2020, with approximately 4.7 million confirmed cases and more than 33,000 deaths reported [11, 12]. Thailand’s COVID-19 vaccination campaign was initiated in March 2020 [11, 13]. Given the emergence of the Delta and Omicron variants between 2021 and 2022, the necessity of administering booster doses of the COVID-19 vaccine has been extensively discussed [13–15]. The uptake rate of COVID-19 vaccines among Thai people was more than 80% [14, 16, 17], likely influenced by the availability of multiple brands of vaccine [14]. Recently, COVID-19 vaccines, including mRNA vaccines (Pfizer and Moderna), viral vector vaccine (AstraZeneca), and inactivated vaccines (Sinovac and Sinopharm), have been implemented for preventing and controlling COVID-19 disease in Thailand [11]. In the scope of our study, the 'booster vaccine' was operationally defined as additional doses of the COVID-19 vaccine administered following a primary course. Conversely, the 'regular vaccine' was defined as a COVID-19 vaccine recommended for universal use, contingent upon factors such as age and vaccination history. However, there remains a lack of information concerning the acceptance of booster vaccines among specific groups in Thailand. Conducting relevant studies is crucial to bridge this knowledge gap and provide valuable insights to inform health policy decisions.

Individuals of all age groups are susceptible to contracting COVID-19. Extensive research has been conducted to understand the impact of COVID-19 on different populations. The CDC reported that young people experience lower mortality rates compared with other age groups and typically exhibit mild disease symptoms [2]. During the COVID-19 vaccine rollout in Thailand, a study [18] conducted at a private university revealed that 1.18% of participants (comprising students and staff) tested positive for COVID-19 infection. According to this study, the majority of participants (> 80%) had already received COVID-19 vaccine, and it was observed that most infected persons were unvaccinated. However, comprehensive data regarding the prevalence of COVID-19 infections among Thai university students since the initial spread of the pandemic is limited. Furthermore, it is important to consider the possibility of individuals experiencing reinfection with COVID-19, especially regarding the potential waning of the immune response to COVID-19 vaccines over time [19]. Therefore, it should be kept in mind that COVID-19 can still be transmitted from young people to other age groups and that young people, due to their frequent interactions and often subtle symptoms, could contribute considerably to the spread of the virus [20].

In Thailand, a substantial proportion of the young population and some of the adult population are currently pursuing higher education, with approximately 1.5 million university students in 2021 [21]. The lifestyles of these students are diverse, encompassing unique societal norms and ways of life. The majority of Thai university students reside in dormitories and engage in communal activities, and they spend a large proportion of their time on campus.

Despite having received a primary course of COVID-19 vaccination, COVID-19 transmission may still occur among these students as well as potentially to their family members. Given that university students represent a specific group with high levels of social contact and potentially large numbers of asymptomatic cases, it is crucial to understand their acceptance of booster doses of COVID-19 vaccines. This would support policymakers in making informed decisions regarding the implementation of emergency and regular vaccine policies in the future. This will be beneficial not only to students but also the entire population when health policy is promulgated to reduce disease transmission. Raising public awareness about disease transmission and the importance of vaccination are crucial for successful implementation of health policy. Given the limited availability of data relating to vaccine acceptance, our study aimed to assess booster vaccine acceptance and factors influencing this among Thai university students. We anticipate that the results of this study will provide valuable insights for decision-making and the effective implementation of health policies in Thailand and other countries in the region with similar levels of resource allocation.

## Methods

### Study sites and participants

Between June and September 2022, we conducted a cross-sectional study among Thai university students to explore their attitudes toward booster doses and whether they would accept them. Any individuals enrolled in universities in Thailand, encompassing both undergraduate (Bachelor) and graduate students (Master or PhD), were deemed eligible to participate in the study. All participants were randomly recruited via an anonymous online questionnaire, using the snowball sampling method [22]. The questionnaire was distributed and aimed to collect data from several universities covering four regions (northern, northeast, middle, and southern) of Thailand.

We estimated the sample size required, using the single-proportion formula with finite population correction [23]. We used the n4Studies application to calculate the sample size, based on previous studies [14, 16, 17, 24] and statistical reports [21]. The main parameters were vaccine acceptance (89%) [14] and the number of university students in Thailand (1.5 million) [21]. Our study thus needed a minimum of 151 participants to ensure a representative sample of the entire student population.

### Data collection

The online questionnaire consisted of two main parts. The first part covered participants' general characteristics, including age, gender, area of study, and education level. The second part focused on aspects related to COVID-19 vaccination. The online questionnaire was distributed to instructors or staff members at universities, who then facilitated distribution to students across different classes. Informed consents and information sheets were provided through an online link. Participants were required to read and agree to participate before accessing the online questionnaire.

### Data analysis

Descriptive statistics, including percentages, means, and standard deviations (SD), were used to explore the various factors examined in the study. The study outcomes were centered around examining the acceptance of COVID-19 vaccine booster doses, which was defined as agreeing to receive ≥ 3 doses and the possibility of receiving regular vaccinations (if available) in the future. To assess the relationships between influencing factors and vaccine acceptance, multiple logistic regression analysis was conducted, estimating odds ratios (ORs) and 95% confidence intervals (CIs). Statistical significance was determined by considering p-values < 0.05 as indicative of significance. Additionally, the Hosmer–Lemeshow goodness-of-fit test provided a systematic way to evaluate the appropriateness of a logistic regression model [25, 26]. The test assesses whether or not the observed event rates match expected event rates in subgroups of the model population. The p-values > 0.05 suggested that the regression model adequately fitted to the data.

## Results

### Participants’ general characteristics

In total, 322 participants provided responses for this study. Most were female (78.9%), and the age range was 18 to 49 years (mean ± SD 22.6 ± 5.47). The majority of participants (85.7%) were enrolled as undergraduate students, with a significant proportion (84.8%) having a background in health sciences studies (Table 1).

**Table 1:** General characteristics of participants (n = 322)

Factor	Details	Frequency	%
Gender	Male	68	21.1
	Female	254	78.9
Age group (years)	18–25	263	81.7
	26–33	43	13.4
	34–41	12	3.7
	42–49	4	1.2
Age range (years)	18–49	322	100.00
Mean age ± SD	22.6 ± 5.47		
Education level	Undergraduate (Bachelor)	276	85.7
	Graduate (Master or PhD)	46	14.3
Study background	Health sciences	273	84.8
	Non-health sciences	49	15.2
Vaccine received (doses)	1	1	0.3
	2	83	25.8
	3	152	47.2
	4	73	22.7
	5	13	4.0
School or job mandate for vaccination	No	35	10.9
	Yes	287	89.1
Booster dose acceptance (willing to receive in the near future)	No	25	7.8
	Not sure	127	39.4
	Yes	170	52.8
Regular vaccine acceptance (if available)	No	26	8.0
	Not sure	73	22.7
	Yes	223	69.3

In terms of vaccination status, the majority of participants had already received the complete standard vaccination course consisting of two doses. The range of COVID-19 vaccine doses administered varied from one to five doses. Specifically, 47.2% of participants had received a third dose, 25.8% had received a second dose, 22.7% had received a fourth dose, and 4.0% had received a fifth dose; just 0.3% had only received one dose. Most of the participants (89.1%) indicated that they were required by their educational institution or place of employment to have the COVID-19 vaccine. The proportions of participants hesitant to receive further COVID-19 booster doses and regular vaccines (if available) were 39.4% and 22.7%, respectively. Only a small proportion of students (7.8% for the booster dose and 8.0% for the regular vaccine) refused to receive the vaccination. Of note, the acceptance rate for booster doses was approximately 52.8%, while the acceptance rate for regular vaccines (if available) was 69.3% (Table 1).

All 322 participants responded to questions about the nine factors that would affect their decisions about whether they would be willing to receive booster vaccines in the near future. More than 80% of participants indicated that all nine of the factors would influence their decision. In the order of magnitude, 98.1% were influenced by vaccine efficacy, 97.8% by product safety, 95.3% by social responsibility, 94.4% by side effects, 94.1% by future career requirements, 93.2% by scientific evidence, 89.8% by vaccine accessibility, 87.6% by previous experiences with COVID-19, and 83.5% by vaccine costs (Table 2).

**Table 2:** Factors influencing participants’ decision whether to have booster vaccines (n = 322)

Which factors influence you with regard to having a vaccination?	Answer	Frequency	%
Vaccine accessibility	No	33	10.2
	Yes	289	89.8
Side effects	No	18	5.6
	Yes	304	94.4
Product safety	No	7	2.2
	Yes	315	97.8
Vaccine costs	No	53	16.5
	Yes	269	83.5
COVID-19 infection history or close contact with COVID-19 patients	No	40	12.4
	Yes	282	87.6
Social responsibility	No	15	4.7
	Yes	307	95.3
Scientific evidence	No	22	6.8
	Yes	300	93.2
Vaccine efficacy	No	6	1.9
	Yes	316	98.1
Future career requirements	No	19	5.9
	Yes	303	94.1

### Factors associated with the acceptance of COVID-19 vaccine booster doses

In the univariate analysis, two factors were significantly associated with the acceptance of COVID-19 vaccine booster doses: vaccine accessibility (crude OR = 3.35, 95% CI = 1.51–7.47) and vaccine costs (crude OR = 1.89, 95% CI = 1.04–3.45). In the context of the multivariate analysis, an adjusted model was constructed to collectively include all relevant factors, with the purpose of simultaneously analyzing individual decision-making processes. Consequently, the adjusted model indicated that vaccine accessibility (adjusted OR = 2.77, 95% CI = 1.10–6.97) was significantly associated with the acceptance of COVID-19 vaccine booster doses (p < 0.05). The Hosmer and Lemeshow goodness-of-fit test revealed a good fit with the data (p = 0.36) (Table 3).

**Table 3:** Factors associated with the acceptance of COVID-19 vaccine booster doses (n = 322)

Factor	Crude OR	95%CI	Adjusted OR	95%CI	p-value
Gender					
Female	1.00 (reference)		1.00 (reference)		
Male	1.47	0.85–2.54	1.47	0.80–2.70	0.22
Education level					
Undergraduate (Bachelor)	1.00 (reference)		1.00 (reference)		
Graduate (Master or PhD)	1.14	0.61–2.13	1.37	0.69–2.72	0.38
Study background					
Health sciences	1.00 (reference)		1.00 (reference)		
Non-health sciences	1.087	0.59–2.00	1.159	0.62–2.18	0.65
School or job mandate					
No	1.00 (reference)		1.00 (reference)		
Yes	1.57	0.77–3.18	1.43	0.66–3.10	0.37
Vaccine accessibility					
No	1.00 (reference)		1.00 (reference)		
Yes	3.35	1.51–7.47	2.77	1.10–6.97	0.03^a^
Side effects					
No	1.00 (reference)		1.00 (reference)		
Yes	1.13	0.43–2.91	0.61	0.26–2.17	0.52
Product safety					
No	1.00 (reference)		1.00 (reference)		
Yes	2.86	0.55–14.95	2.95	0.36–24.46	0.61
Vaccine costs					
No	1.00 (reference)		1.00 (reference)		
Yes	1.89	1.04–3.45	1.53	0.78–3.03	0.21
COVID-19 infection history or close contact with COVID-19 patients					
No	1.00 (reference)		1.00 (reference)		
Yes	1.27	0.66–2.47	0.82	0.42–1.99	0.82
Social responsibility					
No	1.00 (reference)		1.00 (reference)		
Yes	1.29	0.46–3.66	0.96	0.26–3.59	0.96
Scientific evidence					
No	1.00 (reference)		1.00 (reference)		
Yes	1.67	0.69–4.03	1.57	0.55–4.39	0.40
Efficacy					
No	1.00 (reference)		1.00 (reference)		
Yes	1.12	0.22–5.64	0.62	0.08–5.55	0.67
Future career requirements					
No	1.00 (reference)		1.00 (reference)		
Yes	0.80	0.31–2.05	0.48	0.15–1.52	0.21

^a^Significant value (p < 0.05), *OR* odds ratio, *CI* confidence interval

### Factors associated with the acceptance of regular vaccines (if available in the future)

In the univariate analysis, two factors were significantly associated with the acceptance of regular vaccines. These factors included vaccine accessibility (crude OR = 2.34, 95% CI = 1.13–4.84) and the availability of scientific evidence (crude OR = 3.59, 95% CI = 1.48–8.72). In the multivariate analysis, an adjusted model was constructed, similar to the previous multivariate model for COVID-19 vaccine booster doses. This result showed that the availability of scientific evidence (adjusted OR = 3.44, 95% CI = 1.21–9.77) was significantly associated with the acceptance of regular vaccines. The Hosmer and Lemeshow goodness-of-fit test also revealed a good fit with the data (p = 0.64) (Table 4).

**Table 4:** Factors associated with the acceptance of regular vaccines (if available in the future) (n = 322)

Factor	Crude OR	95%CI	Adjusted OR	95%CI	p-value
Gender					
Female	1.00 (reference)		1.00 (reference)		
Male	1.30	0.71–2.37	1.53	0.71–3.03	0.22
Education level					
Undergraduate (Bachelor)	1.00 (reference)		1.00 (reference)		
Graduate (Master or PhD)	1.24	0.64–2.40	1.56	0.75–3.27	0.24
Study background					
Health sciences	1.00 (reference)		1.00 (reference)		
Non-health sciences	1.11	0.58–2.13	1.24	0.63–2.46	0.52
School or job mandate					
No	1.00 (reference)		1.00 (reference)		
Yes	1.20	0.57–2.52	1.16	0.51–2.66	0.73
Vaccine accessibility					
No	1.00 (reference)		1.00 (reference)		
Yes	2.34	1.13–4.84	1.96	0.80–4.79	0.14
Side effects					
No	1.00 (reference)		1.00 (reference)		
Yes	2.38	0.91–6.19	1.87	0.63–5.17	0.28
Safety					
No	1.00 (reference)		1.00 (reference)		
Yes	3.09	0.68–14.06	2.31	0.25–15.09	0.41
Vaccine costs					
No	1.00 (reference)		1.00 (reference)		
Yes	1.46	0.79–2.69	1.09	0.55–2.25	0.81
COVID-19 infection history or close contact with COVID-19 patients					
No	1.00 (reference)		1.00 (reference)		
Yes	1.25	0.62–2.51	0.95	0.41–2.24	0.92
Social responsibility					
No	1.00 (reference)		1.00 (reference)		
Yes	1.13	0.38–3.41	0.35	0.08–1.68	0.27
Scientific evidence					
No	1.00 (reference)		1.00 (reference)		
Yes	3.59	1.48–8.72	3.44	1.21–9.77	0.02^a^
Efficacy					
No	1.00 (reference)		1.00 (reference)		
Yes	4.65	0.84–25.84	6.04	0.54–67.43	0.14
Future career requirements					
No	1.00 (reference)		1.00 (reference)		
Yes	0.58	0.19–1.81	0.20	0.04–1.07	0.20

^a^Significant value (p < 0.05), *OR* odds ratio, *CI* confidence interval

## Discussion

Overall, a total of 322 participants expressed their willingness to participate in our study. Among these participants, a majority were female (78.9%), undergraduate students (85.7%), and possessed a background in health sciences (84.8%). The participants’ ages ranged from 18 to 49 years (mean 22.6, SD ± 5.47). All of them had already been vaccinated, with 99.97% having received a primary course, and more than 70% having received booster doses (≥ 3 doses). The proportion of participants who were hesitant about COVID-19 vaccine booster doses was 39.4%. The proportion of participants who were hesitant about regular vaccines was 22.7%. However, the proportion of students who ultimately chose not to receive a booster dose was relatively small. Our study results revealed the relatively low acceptance rate of COVID-19 vaccine booster doses (52.8%). The value increased slightly to 69.3% when being treated as regular booster doses (Table 1). In our multivariate analysis, we proposed an adjusted model that included all relevant factors that might influence an individual’s decision whether to have a COVID-19 vaccine booster dose. The results revealed that vaccine accessibility (adjusted OR = 2.77, 95% CI = 1.10–6.97) was significantly associated with individuals accepting COVID-19 vaccine booster doses, while the availability of scientific evidence (adjusted OR = 3.44, 95% CI = 1.21–9.77) was significantly associated with individuals accepting regular vaccine doses (Tables 3, 4).

In similar studies around the world, varying proportions of acceptance of booster vaccines among university students have been reported, including 86.3% in Germany [27], 76.2% in Poland [28], and 67.2% in Bangladesh [29]. In comparison, the acceptance of booster doses observed in our study was relatively low. It was also lower than the acceptance of a primary course reported among diverse population groups in Thailand, which were all more than 80% [14, 16, 17]. It is important to note that the differing proportions of acceptance between our study and previous studies may be due to differences in the study contexts. These contextual differences encompass factors such as variations in participant demographics, variations in vaccine choices as well as other relevant national policies at a specific time point.

During the COVID-19 pandemic between 2020 and 2022, several studies emphasized the importance of initial vaccinations against COVID-19, which contributed to reducing infections and disease severity [2, 15, 30]. However, our study was conducted during the period when COVID-19 cases were declining, and the vaccine booster doses had become widely available. The demand for booster doses is likely influenced by the disease situation at the time a survey. The effective management of the COVID-19 pandemic resulting in reduced case numbers [12], could account for the observed decline in booster vaccine acceptance among the participants.

The acceptance of COVID-19 booster doses is subject to a diverse range of influencing factors, encompassing apprehensions regarding uncertainties related to the duration of protection, potential asymptomatic transmission, short- and long-term side effects, and the overall efficacy of the vaccines [24, 30–33]. The existence of these uncertainties contributes to vaccine hesitancy and affects individuals’ willingness to accept booster doses. Discussions and ongoing research regarding the vaccination have remained at the forefront during the pandemic, presenting continuous challenges and considerations for individuals worldwide in their decision-making processes. Additionally, disparities in vaccine availability and access among diverse populations have been observed worldwide [34, 35]. In the context of Thailand, during the initial stages of the COVID-19 vaccine campaign, there were considerable challenges in terms of inadequate vaccine supplies, resulting in limited availability of appointments to receive a vaccine and delays in the vaccination process. Consequently, the target numbers of vaccines allocated to specific regions were not met, prompting individuals to seek vaccination services in different provinces [36, 37]. To address these issues, an easy-to-access vaccine strategy has proven effective in reducing barriers to vaccination, particularly in rural areas [35]. Given these circumstances, it is reasonable to consider the influence of vaccine accessibility on individuals’ intentions to receive booster doses in the future.

Similarly, scientific evidence in support of vaccination played a critical role in various aspects of COVID-19 vaccine acceptance. It served as a crucial foundation for building trust among people, informing decision-making, and promoting vaccine acceptance. It also provided valuable data on vaccine safety and efficacy, established expert consensus, addressed misinformation, facilitated risk–benefit assessments, and enabled effective communication and education [38–40]. This emphasis on seeking valid information and evidence-based decision-making contributed to the overall effectiveness of vaccination strategies in safeguarding public health. Comprehensive, scientifically robust information allowed individuals and policymakers to make informed decisions pertaining to COVID-19 vaccines.

Vaccination costs, including both direct and indirect costs such as vaccine price, hospital fee, and travel cost, were not found to be statistically significant in the multivariate analysis; however, they still remain an important factor to consider. The crude analysis of vaccine costs indicated they did have some impact (OR = 1.89, 95% CI = 1.04–3.45). We considered that the costs of vaccines could potentially act as a financial barrier to vaccination. This was particularly relevant for individuals who experience financial constraints or have a low household income [41–43]. Vaccination costs might increase disparities in access and raise concerns about equity and fairness. A study in Thailand reported that 38.4% of participants were willing to pay for COVID-19 vaccine booster doses [42]. The finding was similar to that of a study conducted in Ethiopia, which found 36.9% of participants were willing to pay for vaccination [44]. These findings indicate the salience of vaccine affordability as a determinant in individuals’ decision-making regarding vaccination. The costs of vaccination could impact individuals’ willingness and ability to seek vaccination services. Furthermore, the absence of vaccination costs may serve as a motivating factor for obtaining booster doses [45], particularly for students with limited financial resources. Consequently, if booster doses are easily accessible without additional charges, it could result in increased vaccination rates and contribute to the containment of disease transmission in the future.

### Limitations

This study had some limitations. The online survey lacked the capacity to comprehend in-depth responses, potentially leading to missed opportunities for gaining nuanced insights into participants' perspectives. Additionally, the findings might not fully reflect the evolving attitudes and behaviors among university students in response to the changing COVID-19 situation and vaccine landscape. In addition, factors influencing vaccine acceptance among Thai university students may change over time. We could not completely ignore the possibilities of duplication when someone attempted to enter their data more than one time, however, we expected a minimal issue as there was no incentive for data providing in this study.

## Conclusion

In conclusion, this study focused on university students in Thailand to address the knowledge gap regarding COVID-19 vaccination among this population, considering their unique lifestyle and potential for asymptomatic transmission. As expected, the study revealed a relatively low level of booster dose acceptance (52.8%) among university students in the country. Our findings suggested that certain factors, such as vaccine accessibility and the availability of scientific evidence, influenced individuals' decisions regarding vaccination. Further research could explore various directions, including studying vaccine acceptance over time, conducting qualitative analyses among different generations, examining geographical distribution, or studying specific population groups. Conducting additional studies will deepen our understanding of vaccine acceptance dynamics and aid in developing targeted strategies to enhance vaccination uptake in the future.

## Data Availability

The data that support the findings of this study are available from the corresponding author, (WP), upon reasonable request.

## Abbreviations

AOR: Adjusted odds ratio
CDC: Center of Disease prevention and Control
CI: Confidence interval
COVID-19: Coronavirus disease 2019
OR: Odds ratio
SD: Standard deviation
WHO: World Health Organization
